# Genome-scale constraint-based modeling of *Geobacter metallireducens*

**DOI:** 10.1186/1752-0509-3-15

**Published:** 2009-01-28

**Authors:** Jun Sun, Bahareh Sayyar, Jessica E Butler, Priti Pharkya, Tom R Fahland, Iman Famili, Christophe H Schilling, Derek R Lovley, Radhakrishnan Mahadevan

**Affiliations:** 1 Genomatica Inc., 10520 Wateridge Circle, San Diego, CA, USA; 2 Department of Chemical Engineering and Applied Chemistry, University of Toronto, Toronto, Ontario, Canada; 3 Department of Microbiology, University of Massachusetts, Amherst, MA, USA

## Abstract

**Background:**

*Geobacter metallireducens *was the first organism that can be grown in pure culture to completely oxidize organic compounds with Fe(III) oxide serving as electron acceptor. *Geobacter *species, including *G. sulfurreducens *and *G. metallireducens*, are used for bioremediation and electricity generation from waste organic matter and renewable biomass. The constraint-based modeling approach enables the development of genome-scale *in silico *models that can predict the behavior of complex biological systems and their responses to the environments. Such a modeling approach was applied to provide physiological and ecological insights on the metabolism of *G. metallireducens*.

**Results:**

The genome-scale metabolic model of *G. metallireducens *was constructed to include 747 genes and 697 reactions. Compared to the *G. sulfurreducens *model, the *G. metallireducens *metabolic model contains 118 unique reactions that reflect many of *G. metallireducens*' specific metabolic capabilities. Detailed examination of the *G. metallireducens *model suggests that its central metabolism contains several energy-inefficient reactions that are not present in the *G. sulfurreducens *model. Experimental biomass yield of *G. metallireducens *growing on pyruvate was lower than the predicted optimal biomass yield. Microarray data of *G. metallireducens *growing with benzoate and acetate indicated that genes encoding these energy-inefficient reactions were up-regulated by benzoate. These results suggested that the energy-inefficient reactions were likely turned off during *G. metallireducens *growth with acetate for optimal biomass yield, but were up-regulated during growth with complex electron donors such as benzoate for rapid energy generation. Furthermore, several computational modeling approaches were applied to accelerate *G. metallireducens *research. For example, growth of *G. metallireducens *with different electron donors and electron acceptors were studied using the genome-scale metabolic model, which provided a fast and cost-effective way to understand the metabolism of *G. metallireducens*.

**Conclusion:**

We have developed a genome-scale metabolic model for *G. metallireducens *that features both metabolic similarities and differences to the published model for its close relative, *G. sulfurreducens*. Together these metabolic models provide an important resource for improving strategies on bioremediation and bioenergy generation.

## Background

*Geobacter *species are environmentally significant because of their capacity for dissimilatory Fe(III) reduction [1]. They can conserve energy for growth by completely oxidizing organic compounds to carbon dioxide coupled to Fe(III) reduction and have been found to be ubiquitous in subsurface environments [2-6]. In addition to Fe(III) reduction, *Geobacter *species can also reduce a variety of toxic and radioactive metals, thus can be applied to efficient bioremediation of uranium, plutonium, technetium, and vanadium [7-9]. *Geobacter *species can also transfer electrons to electrodes to conserve energy for growth [10,11]. It has been demonstrated that *Geobacter sulfurreducens *produces electrically conductive pili that function as nanowires to promote electron transfer to insoluble electron acceptors such as Fe(III) oxide and electrodes [12,13]. Therefore, *Geobacter *species have been utilized to harvest electricity from waste organic matter [10,14] and as a biocatalyst in microbial fuel cell applications [15,16].

*Geobacter metallireducens *was the first organism in pure culture that could oxidize organic compounds with Fe(III) oxide serving as electron acceptor [2,17]. This strict anaerobe can utilize a wide range of organic compounds as electron donors, including acetate, ethanol, propionate, butyrate, pyruvate, propanol, and butanol [2]. More importantly, *G. metallireducens *was found to completely oxidize monoaromatic compounds such as toluene, phenol, cresol, benzoate, benzaldehyde, and benzylalcohol coupled to Fe(III) reduction [2,18]. Several recent studies suggested a core benzoyl-CoA degradation pathway in the utilization of these aromatic compounds [19-22]. *G. metallireducens *can also use nitrate as electron acceptor [2,23].

Constraint-based modeling enables the development of genome-scale *in silico *models that can predict the behavior of complex biological systems and their responses to the environments. Such a modeling approach was successfully applied to provide physiological and ecological insights on the metabolism of *G. sulfurreducens *[24], and has been used to optimize its applications in energy production and bioremediation [25]. Due to its wide range of electron donors and acceptors, *G. metallireducens *has more metabolic capabilities and therefore more potential applications than *G. sulfurreducens*. The genome sequence of *G. metallireducens *was recently completed . Here, we report the development of a genome-scale metabolic model of *G. metallireducens *and the application of the model to study its metabolism.

## Methods

### Metabolic network reconstruction

The *G. metallireducens *metabolic network was reconstructed by a modified version of previously published procedure [26]. The reconstruction was carried out in SimPheny (Genomatica, Inc., CA) from the annotated open reading frames (ORFs) encoded in the *G. metallireducens *genome. The sequence similarity search (BLAST) results of the *G. metallireducens *genome with the genomes of several high-quality genome-scale metabolic models were utilized to create a draft model that served to accelerate the reconstruction of the genome-scale metabolic model. The reactions and genes in the draft model were manually reviewed using the gene annotations and the available biochemical and physiological information. The biomass demand reaction based on biomass composition and maintenance parameters in the published *G. sulfurreducens *model were used in the reconstructed *G. metallireducens *model. The resulting network was then subjected to the gap filling process to allow biomass formation under physiological growth conditions. For gap filling, simulations were performed to determine if the network could synthesize every single component of the biomass and the missing reactions in the pathways were identified. These reactions were reviewed for gene association, or added as non-gene associated reactions to enable the formation of biomass by the reconstructed network under physiological conditions. The reconstructed model was then used to generate a set of experimentally testable hypotheses and predictions. The experimental findings were in turn used to further refine and expand the reconstructed model in an iterative process.

### *In silico *analysis of metabolism

The metabolic capabilities of the *G. metallireducens *model were calculated using flux balance analysis through linear optimization [26] in SimPheny. For growth simulations, biomass synthesis was selected as the objective function to be maximized. For energy requirement simulations, the ATP maintenance requirement reaction was selected as the objective function to be maximized. The simulations resulted in flux values in unit of mmol/g dry weight (gdw)/h. All simulations were of anaerobic growth on minimal media, where the following external metabolites were allowed to freely enter and leave the network: CO_2_, H^+^, H_2_O, K^+^, Mg^2+^, NH_4_^+^, PO_4_^3-^, and SO_4_^2-^. The electron donors or electron acceptors tested were allowed a maximum uptake rate into the network as specified in the results. All other external metabolites were only allowed to leave the system. Flux variability analysis was carried out in SimPheny using method described before [27].

*In silico *deletion analysis was carried out for growth with acetate as the electron donor and Fe(III) or fumarate as the electron acceptor with acetate as the limiting nutrient. Maximization of biomass synthesis was the objective function. Deletions resulting in reduced growth compared to wild type were categorized as intermediate phenotype.

### Strains and culture conditions

The *G. metallireducens *strain used in the growth experiments was constructed with a dicarboxylic acid transporter from *G. sulfurreducens *that grew with fumarate as the sole electron acceptor [28]. The strain was cultured with appropriate electron donors in the NBAF medium that contained 4.64 g/l fumaric acid, 0.42 g/l KH_2_PO_4_, 0.22 g/l K_2_HPO_4_, 0.20 g/l NH_4_Cl, 0.38 g/l KCl, 0.36 g/l NaCl, 0.04 g/l CaCl-H_2_2O, 0.12 g/l MgSO_4_-7H_2_O, 1.8 g/l NaHCO_3_, 0.5 g/l Na_2_CO_3_-H_2_O, and 1 μM Na_2_SeO_4_. The NBAF medium was supplemented with 15 ml/l vitamin mixtures and 10 ml/l mineral mixtures [29,30], and was adjusted to pH 7.0. For growth experiments, the electron donors were added separately from the prepared stocks to the NBAF medium. Final electron donor concentration in the NBAF medium was fixed to 25 mM for both ethanol and pyruvate.

Stock solutions of ethanol and pyruvate were prepared, filtered with 0.2 μm filters, bubbled with N_2_, and capped separately. For the growth experiments, serum bottles containing the culture medium were flushed with N_2_:CO_2 _(80/20) to remove any trace of oxygen in the bottles, capped with thick butyl-rubber stoppers, and autoclaved.

For growth with benzoate, *G. metallireducens *cultures were grown in triplicate at 30°C in anaerobic continuous culture vessels as previously described [30]. Defined, bicarbonate-buffered media with 1.0 mM benzoate as the limiting electron donor and Fe(III) citrate as the electron acceptor was provided at a dilution rate of 0.05 h^-1^. At steady state, protein concentration was 8.2 (± 0.2) mg/L and Fe(II) concentration was 30.3 (± 0.4) mM. Fe(II) was determined using the ferrozine assay as previously described [31].

### Analytical techniques

Samples for organic acid analysis were filtered using 0.2 μm filters and stored at -20°C. The samples were analyzed together using an HPLC (Dionex, Sunnyvale, CA) with a mobile phase of 0.5 mM H_2_SO_4 _at a flow rate of 0.3 ml/min. Peaks were identified and quantified by comparing to those obtained from the standards of ethanol, pyruvate, and fumarate. HPLC data were used to estimate the time profiles of the electron donor and electron acceptor concentrations in samples of *G. metallireducens *in NBAF media.

## Results and discussion

### Metabolic network reconstruction

A draft model of *G. metallireducens *was built by using pair-wise BLASTp comparison of the *G. metallireducens *genome with the genomes of the several high-quality base models in Genomatica model database including previously published *G. sulfurreducens *[24], *Escherichia coli *[26,32,33]and *Bacillus subtilis *[34] models. The *G. metallireducens *draft model comprised 514 reactions. Among the base models used, *G. sulfurreducens *contributed 93% of the top BLASTp matches; this confirmed the close relationship between these two organisms. The *G. metallireducens *draft model captured significant portions of central metabolism, and the biosynthetic pathways for amino acids, nucleotides, and lipids.

The reactions and their gene associations in the draft model of *G. metallireducens *were evaluated manually based on gene annotations, published biochemical and physiological information, and external references as previously described [35]. The remaining genes were also reviewed for inclusion in the reconstructed network. A biomass demand reaction based on the combination of biomass components that were experimentally determined in *G. metallireducens *and represented in the published *G. sulfurreducens *model [24] was used in *G. metallireducens *model. Similarly, the energy parameters such as growth-associated energy requirements in the published *G. sulfurreducens *model [24] were used in the *G. metallireducens *model for the close relationship between these two organisms.

The unique metabolic capabilities of *G. metallireducens *to degrade monoaromatic compounds were reconstructed in the metabolic model. Monoaromatic compounds such as toluene, phenol, cresol, benzoate, benzaldehyde, and benzylalcohol are converted into benzoyl-CoA and then through the benzoyl-CoA degradation pathway to acetyl-CoA [19-22]. Specifically, benzylalcohol and benzaldehyde are oxidized by dehydrogenases to benzoate, which is then converted into benzoyl-CoA by benzoate CoA ligase, whereas cresol and phenol are converted to 4-hydroxybenzoate and then reduced to benzoyl-CoA through 4-hydroxybenzoyl-CoA. Toluene is converted to benzoyl-CoA via benzylsuccinyl-CoA.

For gap filling, the ability of the metabolic network to synthesize a full complement of amino acids, nucleotides, lipids, carbohydrates, and cofactors from a minimal medium containing the known electron donors and acceptors was assessed. The missing reactions in the pathways were identified and reviewed. Some missing reactions were associated with *G. metallireducens *genes based on biochemical or genomic evidences and were included in the reconstructed network. Other missing reactions were added to the model as non-gene associated reactions to enable the reconstructed network to synthesize metabolites for biomass formation. The reconstructed network contains 30 non-gene associated reactions with different justification. These non-gene associated reactions fell into several categories: 2 reactions, 2-Oxo-4-methyl-3-carboxypentanoate decarboxylation and L-glutamate 5-semialdehyde dehydratase, are non-enzymatic conversions that happen spontaneously under physiological conditions; 4 gas diffusion processes allow the transport of these gases; 1 reaction is for ATP maintenance requirement; 5 transporter reactions for electron donors ensure consistency with growth results; and 18 non-gene associated reactions are required for biomass formation under known growth conditions (see Additional file 1 for details). Non-gene associated reactions in the latter two categories are presumptive metabolic functions encoded potentially by unknown genes, and thus will be subjected for further genomic and biochemical investigation in the future.

**Figure 1:**
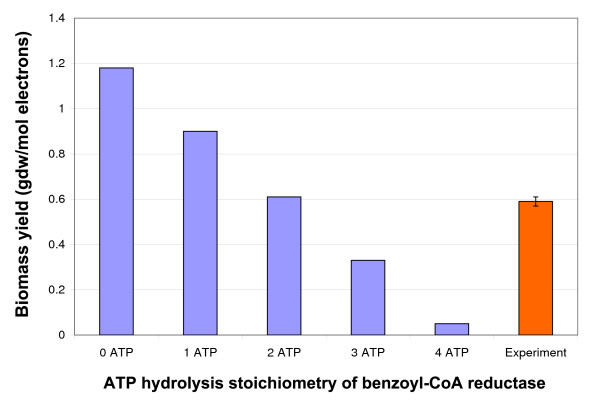
**ATP hydrolysis stoichiometry of benzoyl-CoA reduction in *G. metallireducens *metabolic model**. Benzoyl-CoA reductase reactions with 0–4 ATP hydrolysis stoichiometry were applied in simulations. The predicted biomass yields as gdw/mol electrons were calculated and compared to the experimental result obtained from protein content measurement at steady state.

Simulations were also utilized to understand individual reactions in the network. For example, the initial step of benzoyl-CoA degradation pathway is catalyzed by a benzoyl-CoA reductase. In *Thauera aromatica*, benzoyl-CoA reductase reduces the aromatic ring in two single-electron transfer steps to yield cyclohexa-1,5-diene-1-carbonyl-CoA with stoichiometric 2-ATP hydrolysis [36]. To understand the ATP hydrolysis stoichiometry associated with benzoyl-CoA reduction in *G. metallireducens*, biomass was collected from *G. metallireducens *cells grown with benzoate in chemostat and the experimental results were compared to simulation results where different ATP hydrolysis stoichiometry was assumed for the benzoyl-CoA reduction (Figure 1). The experimental growth data, a protein yield of 8.2 (± 0.2) mg/L from 1.0 mM benzoate at a dilution rate of 0.05 h-1, predicted a biomass yield of 0.59 (± 0.02) gdw per mol of electrons at benzoate flux of 2.81 mmol/gdw/h assuming 46% biomass content as protein. The biomass yields from a benzoate flux of 2.81 mmol/gdw/h with an ATP hydrolysis stoichiometry for benzoyl-CoA reduction between 0–4 were simulated and the in silico results were compared to the experimental result (Figure 1). As shown in Figure 1, the 2-ATP hydrolysis stoichiometry for benzoyl-CoA reduction closely matched the experimental result. Thus, the benzoyl-CoA reduction in *G. metallireducens *model shared the same ATP hydrolysis stoichiometry as in *T. aromatica*.

### Metabolic network of *G. metallireducens*

**Figure 2:**
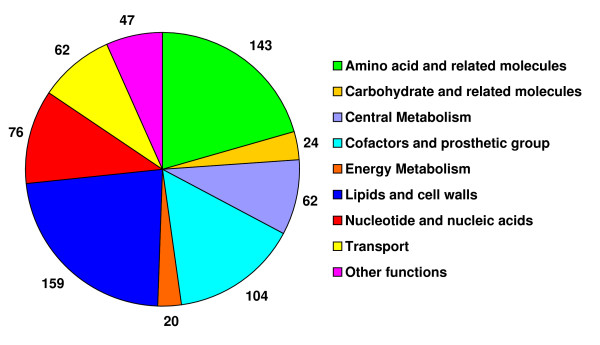
**Functional classification of metabolic reactions in *G. metallireducens *model**. The 697 reactions in *G. metallireducens *model were categorized into 9 functional groups.

At its completion, the manually curated genome-scale network of *G. metallireducens *included 747 genes of the 3389 genes in the *G. metallireducens *genome (Table 1). The *G. metallireducens *metabolic model contains 697 reactions and 769 metabolites including 58 extracellular metabolites. The detailed list of genes, reactions, metabolites, and gene-protein-reaction (GPR) associations in the metabolic model are available as supplementary information (see Additional file 2). The characteristics of the *G. metallireducens *model are similar to those of the updated *G. sulfurreducens *model (the published *G. sulfurreducens *model [24] was updated to incorporate the most recent results from both experimental and computational research, see Additional file 3 for detailed list of reactions). The 697 reactions of the *G. metallireducens *model were categorized into 9 functional groups and the results were summarized in Figure 2. Among different functional groups, reactions for biosynthesis of amino acids, lipids and cell wall components, cofactors, and nucleic acids are the most abundant, accounting for almost 70% of all the reactions. Currently, there are 76 reactions associated with transporting metabolites, including redundant transporters for the some extracellular metabolites. In addition, *G. metallireducens *genome contains many genes encoding components of these ABC transporters that are not included in the network because the substrate specificity of these ABC transporters is largely unknown. Future experiments on physiology in different environments will provide additional evidence to include these transporting systems.

**Table 1:** Characteristics of the *G. metallireducens *genome-scale metabolic model compared with the *G. sulfurreducens *model.

	***G. metallireducens***	***G. sulfurreducens***
**Total Genes**	3532	3468
Included Genes	747 (21.2%)	730 (21.1%)
Excluded Genes	2785 (78.8%)	2738 (78.9%)
**Total Proteins**	623	582
**Total Reactions**	697	649
Non-gene Reactions	30 (3.7%)	32 (4.9%)
Input/Output Reactions	60	55
**Total Metabolites**	769	698
Extracellular Metabolites	58 (7.7%)	55 (7.9%)

To study the conservation between the *G. metallireducens *and *G. sulfurreducens *models, reactions were categorized and compared (Table 2). Overall, the two models share 579 common reactions, representing 83% of all *G. metallireducens *reactions and 89% of all *G. sulfurreducens *reactions. Among these common reactions, 140 reactions related to amino acid biosynthesis, 119 reactions in lipids and cell walls metabolism, 104 reactions of cofactor biosynthesis, and 74 reactions for nucleotide metabolism are shared between the two models, which together account for 75% of all the common reactions.

**Table 2:** Comparison of reactions in *G. metallireducens *and *G. sulfurreducens *metabolic models.

	***G. sulfurreducens***			***G. metallireducens***			
	All	Unique	Percentage	All	Unique	Percentage	Common
Amino acid and related molecules	148	8	5.41%	143	3	2.10%	140
Carbohydrate and related molecules	21	4	19.05%	24	7	29.17%	17
Central Metabolism	56	8	14.29%	62	14	22.58%	48
Cofactors and prosthetic group	106	2	1.89%	104	0	0.00%	104
Energy Metabolism	22	5	22.73%	20	3	15.00%	17
Lipids and cell walls	130	11	8.46%	159	40	25.16%	119
Nucleotide and nucleic acids	77	3	3.90%	76	2	2.63%	74
Transport	61	24	39.34%	62	25	40.32%	37
Other functions	28	5	17.86%	47	24	51.06%	23
							
Total reactions	649	70	10.79%	697	118	16.93%	579

*G. metallireducens *can utilize a much wider range of electron donors and acceptors [2,18,23] than *G. sulfurreducens*, which uses only acetate, H_2 _and lactate as the electron donors. The *G. metallireducens *metabolic model contains 118 unique reactions out found in the *G. sulfurreducens *model. Many of these unique reactions reflect of the diversity of *G. metallireducens*' metabolic capabilities. For example, the *G. metallireducens *model contains 32 unique reactions involved in the degradation pathways of aromatic compounds. *G. metallireducens *can also utilize several substrates other than the aromatic compounds that *G. sulfurreducens *does not use. *G. metallireducens *contains several alcohol dehydrogenase genes with substrate specificities for ethanol, propanol, and butanol that are not believed to be present in *G. sulfurreducens*. The enzymes coded by these genes catalyze several unique reactions that are key steps in the utilization of these alcohol substrates. The corresponding transporter reactions were also added to the *G. metallireducens *model, but not in the *G. sulfurreducens *model. Similarly, a butyrate kinase reaction unique to the *G. metallireducens *model allows the utilization of butyrate. These unique reactions in the *G. metallireducens *model enable the growth of the *G. metallireducens *model on a wide range of substrates and has accurately captured the known physiological characteristics of *G. metallireducens *[2,18,23].

### *In silico *characterization of *G. metallireducens *metabolism

**Figure 3:**
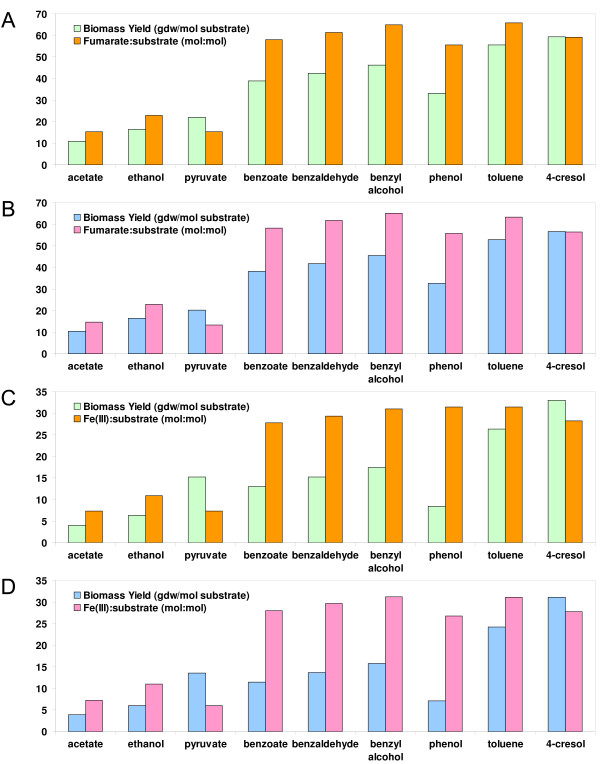
***In silico *characterization of *G. metallireducens *metabolism**. *G. metallireducens *was simulated to grow with 9 different electron donors (acetate, ethanol, pyruvate, benzoate, benzaldehyde, benzyl alcohol, phenol, toluene, and 4-cresol) and 2 electron acceptors [fumarate and Fe(III)]. The biomass yields to substrate (gdw/mol substrate) and the acceptor to substrate ratios (mol:mol) predicted by the model are shown in the figures. ***A***. Fumarate as the electron acceptor under donor limiting conditions; ***B***. Fumarate as the electron acceptor and the limiting nutrient; ***C***. Fe(III) as the electron acceptor under donor limiting conditions; and ***D***. Fe(III) as the electron acceptor and the limiting nutrient.

Simulations of metabolism with the *G. metallireducens *model were utilized to make testable predictions of *G. metallireducens *metabolism. *In silico *characterization of *G. metallireducens *growth with different substrates was carried out and the results are summarized in Figure 3. The growth of *G. metallireducens *was simulated using 9 substrates as electron donors with either Fe(III) or fumarate as electron acceptor and setting the electron donor or electron acceptor as the limiting factor. Under all 4 conditions, 4-cresol provided the largest biomass yield per substrate (calculated as gdw/mol substrate) for *G. metallireducens *growth while acetate produced the lowest biomass yield among the 9 substrates tested. Aromatic compounds generated higher biomass yield per mole of substrate than acetate and ethanol. The biomass yields for the 9 substrates under electron acceptor limiting conditions were similar to those under electron donor limiting conditions (Figure 3A&3B, 3C&3D), suggesting *G. metallireducens *might not fully utilize the excess electron donors under acceptor limiting conditions. During Fe(III) reduction, *G. metallireducens *had similar predicted biomass yields on pyruvate and benzoate. This is because of the energy gain associated with the conversion of pyruvate to acetyl-CoA, whereas benzoate degrades to acetyl-CoA and energy is consumed to convert acetyl-CoA to pyruvate for biomass.

When biomass yields were calculated based on acceptor consumed, pyruvate resulted in the highest biomass yield per mol of electron acceptor under all conditions, suggesting that pyruvate may have advantages over other substrates in electron acceptor limiting environments. Acetate and ethanol had similar biomass yield per electron acceptor compared to the aromatic compounds, suggesting that they may produce the same amount of biomass when limited to same amount of electron acceptors in growth medium. Therefore, the modeling study rapidly predicted the growth yields of *G. metallireducens *under varying nutrient conditions.

### Comparison of *G. metallireducens *metabolic model to *G. sulfurreducens *model

*G. metallireducens *also contains genes for several pathways in central metabolism that do not have corresponding homologues in *G. sulfurreducens*. Therefore, the unique reactions associated with these genes may provide specific metabolic capacities in the *G. metallireducens *model. For example, *G. metallireducens *is known to use nitrate as an electron acceptor [2,23] and the model predicts such capability. The *G. metallireducens *network has transporters for nitrate uptake via nitrite antiport and the nitrate reductase (cytochrome *c*) to reduce nitrate, which are not present in *G. sulfurreducens*. These two reactions together allow electrons from cytochrome *c *to be transferred to nitrate. Nitrate is reduced and the resulting intracellular nitrite is exchanged with extracellular nitrate using the antiporter. The *G. metallireducens *model also contains a nitrite proton antiporter and nitrite reductase that further reduce nitrite to ammonium and allows the utilization of nitrite.

Other reactions that are not present in the *G. sulfurreducens *model include the glucose 6-phosphate dehydrogenase, 6-phosphogluconolactonase, and phosphogluconate dehydrogenase, which is a part of the oxidative branch of the pentose phosphate pathway. This branch provides an efficient way to produce D-ribose-5-phosphate and is an important source of NADPH. However, simulations of *G. metallireducens *growth predict that *G. metallireducens *can produce D-ribose-5-phosphate by using glyceraldehyde 3-phosphate and D-fructose-6-phosphate to produce D-xylulose 5-phosphate through transketolase and transaldolase, and then converting D-xylulose 5-phosphate to D-ribose-5-phosphate, similar as simulation of *G. sulfurreducens *growth. Simulations also suggest that *G. metallireducens *can generate NADPH through isocitrate dehydrogenase (NADP) and other reactions with NADP as cofactor in a manner similar to the *G. sulfurreducens *network. There was no significant change in the expression levels of these genes during growth with acetate vs. benzoate couples with Fe(III) reduction. The exact role of this oxidative branch of pentose pathway in *G. metallireducens *requires further examination.

ATP-consuming futile cycles involve multiple reactions allowing the interconversion between metabolites with a net ATP consumption, and can decrease growth. However, it is hypothesized that these futile cycles balance the metabolite pools to make other key reactions thermodynamically feasible [37]. Recent ^13^C-labeling studies in *G. metallireducens *confirmed the existence of an ATP-consuming futile cycle between pyruvate and phosphoenolpyruvate [37].

**Figure 4:**
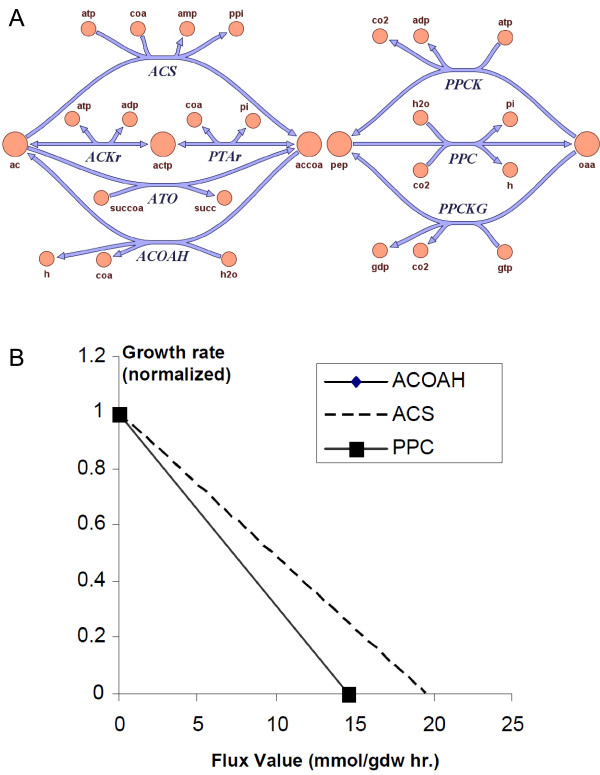
**Potential futile cycles in the central metabolism of *G. metallireducens *model**. ***A***, Two examples of energetically inefficient reactions that can form futile cycles in the central metabolism of *G. metallireducens *model. ***B***, Effect of increasing flux through the energetically inefficient reactions on the growth rate.

The central metabolism of *G. metallireducens *has several reactions that are missing in *G. sulfurreducens*. These reactions include the acetyl-CoA synthetase (ACS), acetyl-CoA hydrolase (ACOAH), and phosphoenolpyruvate carboxylase (PPC) reactions. These reactions may be energetically inefficient because they can participate in futile cycles that drain ATP (Figure 4A). The acetate activation reaction ACS in *G. metallireducens *is energetically inefficient (consuming two ATP equivalents to form one acetyl-CoA), compared to the acetyl-CoA transferase (ATO) and the combined acetate kinase/phosphotransacetylase (ACK/PTA) pathway (consuming one ATP equivalent to form one acetyl-CoA) that are present in both models. The ACOAH reaction produces zero ATP to convert acetyl-CoA to acetate and can form futile cycles with the three routes of acetate activation, namely, the ATO, the ACS and the ACK/PTA pathways. A similar futile cycle involves phosphoenolpyruvate carboxylase and phosphoenolpyruvate carboxykinase allowing ATP-consuming interconversion between phosphoenolpyruvate and oxaloacetate (Figure 4A). Model simulations also predict that increasing fluxes through these reactions result in an energetic penalty and consequently lowered biomass yield (Figure 4B).

### *G. metallireducens *growth with electron donors

**Figure 5:**
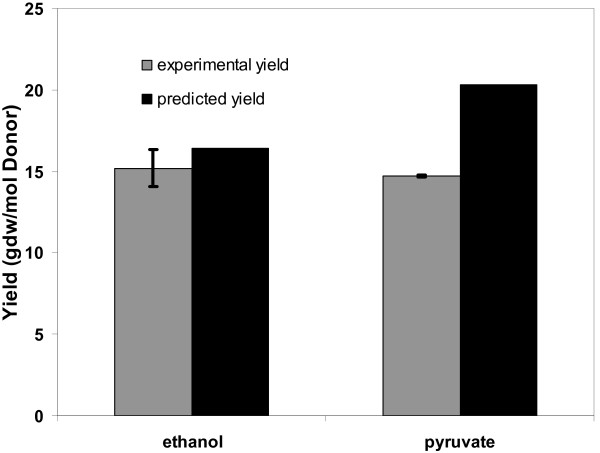
**Comparison of experimental and predicted biomass yields**. The experimental and predicted biomass yields were obtained for growth with fumarate and two different electron donors, namely, pyruvate and ethanol.

In order to further investigate the potential for these energetically inefficient reactions to decrease biomass yields, we measured the experimental growth of *G. metallireducens *in the presence of different electron donors. Unlike *G. sulfurreducens, G. metallireducens *can oxidize ethanol and pyruvate, thus enabling the further investigation of the central metabolism of the *Geobacter *species. In order to isolate the effect of the different electron donor oxidation pathways on the yield, a *G. metallireducens *strain with a dicarboxylic acid transporter that allows growth using fumarate as electron acceptor, was cultured with ethanol and pyruvate as electron donors. The *G. metallireducens *model simulations suggested that the biomass yield to substrate consumed (calculated as gdw/mol substrate) of pyruvate should be 34% higher than that of ethanol (Figure 5). However, the experimental results showed that similar biomass yields were obtained in pyruvate or ethanol cultures (Figure 5). HPLC measurements confirmed the complete utilization of pyruvate (data not shown). These results suggested potential energetic inefficiencies during growth with pyruvate. Most likely, the energy-inefficient reactions discussed above were active and resulted in the decreased biomass yield during growth on pyruvate.

Microarray data for the above growth conditions were not readily available. Instead, we analyzed the microarray data for *G. metallireducens *growing with benzoate versus acetate [21]. Among *G. metallireducens *genes that were significantly up-regulated (> 50%) by growth with benzoate versus acetate, genes encoding for ACS, PPC, and ACOAH were up-regulated by 161% to 270%. The up-regulation of these genes encoding for the energy-inefficient reactions during growth with the complex substrate benzoate indicated the involvement of these energy-inefficient reactions in the metabolism of *G. metallireducens *when high-energy substrate benzoate is consumed. It is likely that similar up-regulation of these genes occurs during growth with pyruvate and not with ethanol.

Simulations of the *G. metallireducens *growth were performed using maximal biomass yield as the objective function, usually to be true in natural growth conditions where nutrients are limited. However, optimizing biomass yield may not always be the growth strategy of choice and recent studies have illustrated that under conditions of nutrient excess, maximizing the ATP production might be the chosen growth strategy [38]. Growth with high-energy substrates may also lead to a growth strategy of maximal ATP production. Under this growth strategy, the energetically inefficient reactions can be advantageous for utilizing the ATP produced. The abundance of these energetically inefficient reactions in *G. metallireducens *suggests that the evolution of *G. sulfurreducens *and *G. metallireducens *might have occurred in environments with different nutrient levels. In this scenario, *G. sulfurreducens *probably evolved in predominantly acetate limiting environments, whereas *G. metallireducens *probably evolved in environments with nutrient excess or with complex nutrients available.

### Growth simulations of *G. metallireducens *using nitrate as electron acceptor

**Figure 6:**
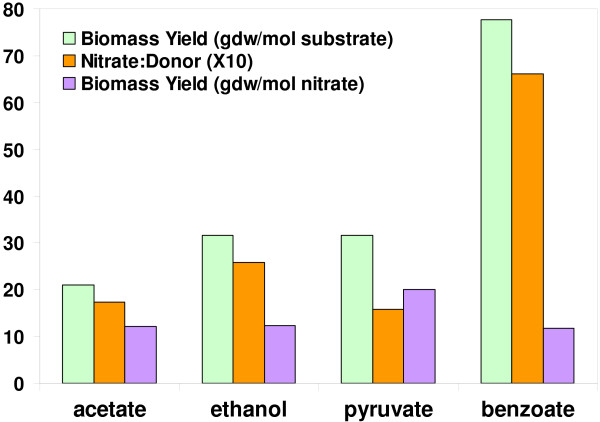
**Simulated growth of *G. metallireducens *with nitrate as electron acceptor**. Growth of *G. metallireducens *was simulated using different electron donors under donor-limiting conditions. Biomass yields were predicted for both electron donor (gdw/mol substrate) and electron acceptor (gdw/mol nitrate). The ratios of nitrate to electron donors are also shown in the figure.

*G. metallireducens *can use nitrate as an electron acceptor [2,23]. To better understand this capacity of nitrate respiration, *G. metallireducens *growth simulations were performed using nitrate as electron acceptor and acetate, ethanol, pyruvate or benzoate as electron donor. As shown in Figure 6, when nitrate was used as acceptor for *G. metallireducens *growth, benzoate was predicted to give the highest biomass yield to substrate (gdw/mol substrate). Pyruvate and ethanol were predicted to equally produce more biomass per substrate consumed than acetate. However, using pyruvate as substrate allowed the lowest acceptor:donor ratio, whereas benzoate had the highest. Pyruvate as substrate was predicted to have the highest biomass yield per nitrate (gdw/mol nitrate) similar to the cases discussed earlier when Fe(III) or fumarate is the electron acceptor.

**Figure 7:**
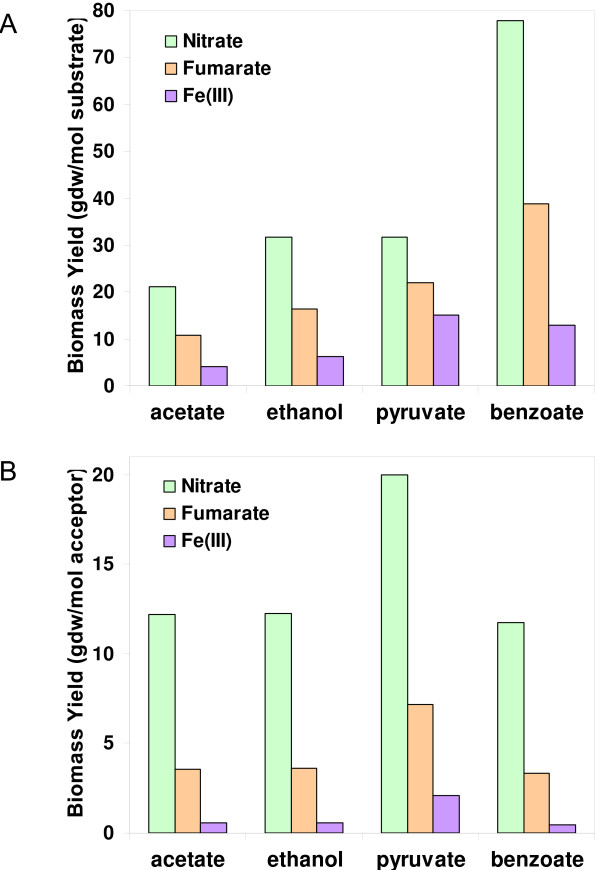
**Comparison of *G. metallireducens *growth with different electron acceptors**. Growth of *G. metallireducens *was simulated with the oxidation of four different electron donors (acetate, ethanol, pyruvate, and benzoate) coupled to the reduction of electron acceptors nitrate, fumarate, and Fe(III) under donor-limiting conditions. *A*. Predicted biomass yields to the electron donors (gdw/mol substrate) with nitrate, fumarate and Fe(III) as the acceptor; *B*. Predicted biomass yields to the electron acceptors (gdw/mol acceptor) during nitrate, fumarate and Fe(III) reduction with different electron donors.

Growth simulations of *G. metallireducens *using nitrate, fumarate or Fe(III) as electron acceptor were compared (Figure 7). Among the three electron acceptors, nitrate resulted in the highest biomass yield per substrate consumed (gdw/mol substrate) or per electron acceptor consumed (gdw/mol electron acceptor). This is consistent with the higher energy yield coupled to nitrate reduction. The large increase in biomass yield during nitrate reduction relative to Fe(III) reduction predicts that *G. metallireducens *is not limited by energy generation during nitrate reduction. For example, the fraction of the benzoate used to generate energy during nitrate reduction was predicted to be 63%, compared to 94% of the benzoate used for generating energy during Fe(III) reduction. This requirement of a relatively high fraction of donor for energy generation results in higher substrate utilization rates for the same growth rate during Fe(III) reduction clearly highlighting a significant challenge associated with metal reduction.

### Flux distribution comparison between model prediction and ^13^C labeling results

**Figure 8:**
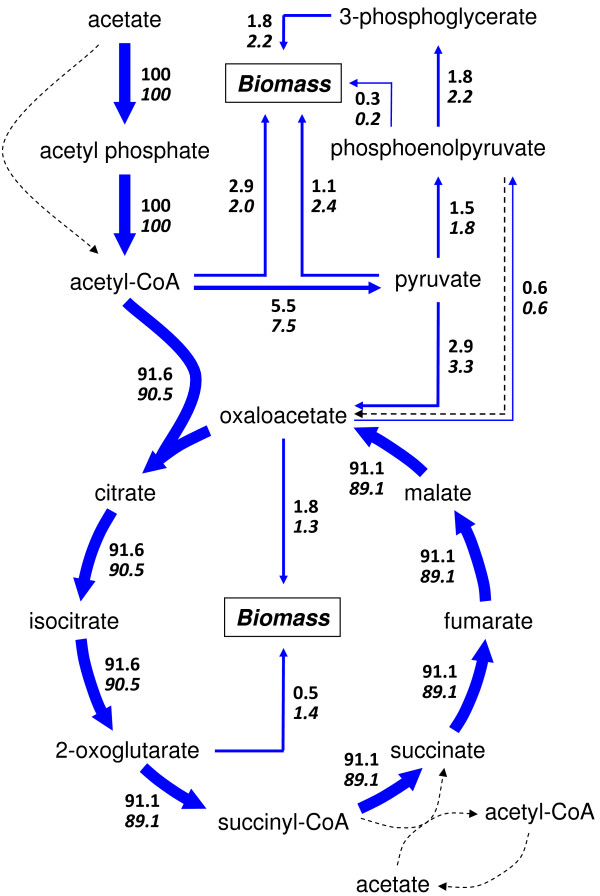
**Metabolic flux distributions in *G. metallireducens *by *in silico *genome-scale modeling**. *G. metallireducens *growth with acetate as electron donor and Fe(III) as electron acceptor was simulated with under 21 mmol/gdw/h of acetate uptake flux with acetate as the limiting factor, the same as a ^13^C isotopomer labeling flux analysis. The predicted flux distributions by the genome-scale modeling (*upper numbers*) were compared to the flux distributions determined in the [1-^13^C]acetate labeling experiments (*lower numbers*). Dotted arrows indicated the pathways involving some energy-inefficient reactions that had no flux under this condition.

^13^C isotopomer labeling flux analysis was applied to study a simplified central metabolic network of *G. metallireducens *by Tang, *et al*. [37]. The results from that study provided an experimentally determined flux distribution. To validate the *G. metallireducens *model reconstructed in this work, the flux distribution results from ^13^C isotopomer labeling flux analysis study was compared to an *in silico *flux distribution from the *G. metallireducens *model under the same acetate/Fe(III) growth conditions (21 mmol/gdw/h of acetate uptake flux with acetate as the limiting factor). Fluxes were normalized according to acetate uptake rate that was set at 100%. The ^13^C isotopomer labeling flux analysis suggested that about 90% of flux from acetyl-CoA joined the TCA cycle to produce energy and 10% of flux was routed to pyruvate and other intermediates for biomass [37]. As shown in Figure 8, model simulations predicted 91.6% of acetate was completely oxidized to CO_2 _via the complete TCA cycle, compared to the 90.5% from the [1-^13^C] acetate isotopomer labeling flux analysis. Overall, the flux distributions were similar (mean of the flux difference = 1% ± 0.8%, R^2 ^= 0.99). The computationally predicted and experimentally determined values were well matched at high fluxes, but less consistent at low fluxes. One difference between the two analysis is that the [1-^13^C] acetate isotopomer labeling flux analysis used a network where serine, glycine, and cysteine were derived from 3-phosphoglycerate. However, gene encoding for these functions has not been found in *G. metallireducens*, whereas genes for a pathway where serine, glycine, and cysteine were derived from oxaloacetate in the TCA cycle were identified and used in *G. metallireducens *genome-scale model and simulations. This may account for some of the differences, such as the differences of fluxes from 3-phosphoglycerate or oxaloacetate to biomass, observed between the model simulation and the ^13^C labeling flux analysis results.

Flux variability analysis defines a feasible range of fluxes for each individual reaction [27]. A flux variability analysis under the same constraints indicated that most flux values determined by ^13^C labeling experiments were within such feasible ranges (data not shown), and validated the consistency between the experimental and predicted results. These results suggested that *in silico *growth simulation optimized for biomass formation and flux variability analysis to define the feasible flux ranges together provided a fast and easy alternative method to estimate flux distribution for the metabolism of *G. metallireducens*.

### Functional analysis of *G. metallireducens *mutant phenotype

**Figure 9:**
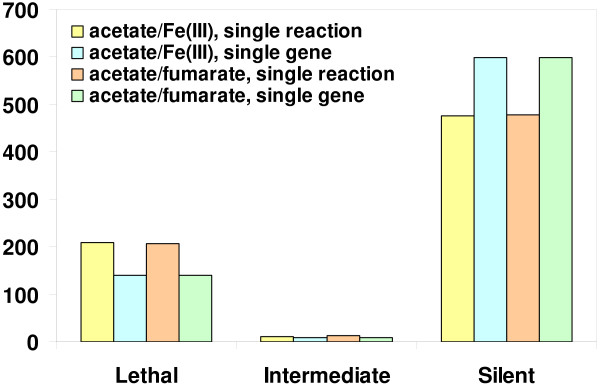
**Functional analysis of *G. metallireducens *mutant phenotype**. *In silico *deletion analyses for *G. metallireducens *growth were completed using electron donor/acceptor pairs of acetate/Fe(III) or acetate/fumarate for every single reaction or every single gene included in the genome-scale metabolic model.

Genome-scale metabolic model enabled the systems level gene deletion analysis for growth in defined medium. This information will provide important insight into the potential phenotypes associated with gene deletions in genetic investigations. *In silico *deletion analyses for *G. metallireducens *growth using electron donor/acceptor pairs of acetate/Fe(III) or acetate/fumarate were completed and the results were shown in Figure 9. Three possible phenotypes were predicted from the deletion analysis: 1) lethal deletion with no growth observed, 2) silent mutation growing same as wild type, and 3) intermediate phenotype with reduced growth. Simulation results were the same when either Fe(III) or fumarate was used as electron acceptor. More than 68% of all reactions or 80% of all included genes were predicted to have no effect on growth upon deletion. About 30% of all reactions and 19% of included genes were lethal mutations reflecting the inability of the perturbed network to synthesize essential components. Only for 1–2% of all reactions and included genes, deletions were predicted to have an intermediate effect on the growth rate of the *G. metallireducens *growth under the conditions. These deletion analysis results are similar to the results from the *G. sulfurreducens *model. This suggests that the core metabolic pathways are conserved among *Geobacteraceae*, and that the models of *G. metallireducens *and *G. sulfurreducens *can be used to represent of the physiology of *Geobacteraceae*.

## Conclusion

Environmental pollution and sustainable energy are among the most important challenges that the world is facing in the 21st century. Consequently, these areas have attracted significant research efforts. In particular, *Geobacter *species are being extensively studied for their applications in bioremediation and bioelectricity production. However, similarities and differences in the metabolism and physiology of *Geobacter *species have not been well characterized. In this report, we have developed a genome-scale metabolic model for *G. metallireducens *to accelerate discovery and gain insight into its metabolism. Together with the published *G. sulfurreducens *model, the *G. metallireducens *metabolic model provides an important resource for the improving strategies for bioremediation and bioenergy generation.

The reconstructed metabolic model of *G. metallireducens *was used to gain insight into the metabolism of this bacterium. The *G. metallireducens *model is metabolically distinct from the *G. sulfurreducens *model, largely due to the wider range of *G. metallireducens *substrate utilization. The *G. metallireducens *metabolic model contains many additional reactions reflecting these specific metabolic capabilities that the *G. sulfurreducens *model does not have. *In silico *modeling of these additional metabolic capabilities can be used to understand how these substrates are utilized by *G. metallireducens *and how these capabilities can be applied in bioremediation and bioelectricity production.

Detailed examination of the *G. metallireducens *model suggested that its central metabolism contains several energy-inefficient reactions that are not present in the *G. sulfurreducens *model. Experimental biomass yield of *G. metallireducens *growing with pyruvate was lower than the predicted optimal *in silico *biomass yield, and microarray data of *G. metallireducens *growing with benzoate and acetate indicated that genes encoding these unique reactions were up-regulated by benzoate. These results suggested that the energy-inefficient reactions were likely turned off during *G. metallireducens *growth with acetate to optimize biomass yield, but were up-regulated during growth with complex electron donors to improve flux for rapid energy generation. Thus, the evolution of *G. sulfurreducens *and *G. metallireducens *might have occurred in environments with different nutrient levels: *G. sulfurreducens *in a predominantly acetate limiting environments, whereas *G. metallireducens *probably in environments in the presence of complex nutrients or nutrient abundance. These results will help understand the physiology of these *Geobacter *species in the subsurface environments.

Furthermore, several *in silico *computational modeling approaches were applied to accelerate *G. metallireducens *research. For example, growth of *G. metallireducens *with different electron donors and electron acceptors were simulated using the genome-scale metabolic model. These simulations provided an easy and cost-effective way further understanding the metabolism of *G. metallireducens*. Flux distribution was compared between *in silico *prediction and ^13^C labeling flux analysis results, suggesting that *in silico *prediction could provide a fast alternative method to estimate metabolic fluxes. Finally, the deletion analysis of the *G. metallireducens *metabolic model predicts phenotypes of gene knock-outs systematically and quickly. It is also important to understand that the testable hypotheses and predictions generated by *in silico *computational modeling with the reconstructed model should be evaluated experimentally. The experimental findings will in turn further refine and expand the reconstructed model, as well as improve our understanding of the *Geobacter *metabolism, in an iterative fashion.

## Abbreviations

ACKr: acetate kinase; ACOAH: acetyl-CoA hydrolase; ACS: acetyl-CoA synthetase; ATO: acetyl-CoA transferase; gdw: gram dry weight; ORF: open reading frame; PTAr: phosphotransacetylase; PPC: phosphoenolpyruvate carboxylase; PPCK: phosphoenolpyruvate carboxykinase; PPCKG: phosphoenolpyruvate carboxykinase (GTP); ac: acetate; accoa: acetyl-CoA; actp: acetyl phosphate; oaa: oxaloacetate; pep: phosphoenolpyruvate; succ: succinate; succoa: succinyl-CoA; ORF: open reading frames; gdw: g dry weight; GPR: gene-protein-reaction.

## Authors' contributions

JS, PP, TRF, and IF developed the genome-scale metabolic model of *G. metallireducens*. BS and JEB carried out the growth experiments. JS and RM analyzed the experimental data and drafted the manuscript. JS, CHS, DRL, and RM conceived the study and revised the manuscript. All authors read and approved the final manuscript.

## Supplementary Material

Additional file 1 **Non-gene associated reactions in the *G. metallireducens *model.** This table provides the detailed information for the non-gene associated reactions in the *G. metallireducens *model, including justification for the inclusion of each non-gene associated reaction in the model. Click here for file

Additional file 2 **All the reactions, genes, and metabolites in the *G. metallireducens *model.** The data provide comprehensive lists of genes, reactions, metabolites, and GPR associations in the *G. metallireducens *metabolic model. Click here for file

Additional file 3 **All the reactions, genes, and metabolites in the *G. sulfurreducens *model.** The data provide comprehensive lists of genes, reactions, metabolites, and GPR associations in the *G. sulfurreducens *metabolic model. Click here for file
